# *Diplycosia platyphylla* (Ericaceae), a new species from Mindanao, Philippines

**DOI:** 10.3897/phytokeys.69.9466

**Published:** 2016-08-18

**Authors:** Peter W. Fritsch, Victor B. Amoroso

**Affiliations:** 1 Botanical Research Institute of Texas, 1700 University Drive, Fort Worth, Texas 76107, USA; 2 Center for Biodiversity Research and Extension in Mindanao, Central Mindanao University, University Town, Musuan 8710, Bukidnon, Philippines

**Keywords:** Diplycosia, Ericaceae, Gaultherieae, Mindanao, Mount Apo, new species, Philippines

## Abstract

*Diplycosia platyphylla* P.W.Fritsch, a new species from Mindanao Island, Philippines, is described and illustrated. This species is most similar to the Bornean *Diplycosia urceolata* but differs by its green or slightly flushed pink petioles 4–7 mm long, wider leaf blades, acute calyx lobe apices, and lavender mature fruiting calyx. The new species is known only from a single collection made from Mount Apo in North Cotabato Province, southern Mindanao.

## Introduction

*Diplycosia* Blume (Ericaceae: Vaccinioideae: Gaultherieae) comprises about 116 species distributed throughout Southeast Asia and New Guinea (Sleumer 1967; Argent 1982, 1989, 2002; Ferreras and Argent 2011; Argent 2013, 2014; Fritsch and Amoroso in press; Fritsch and Bush in press). The genus can be delimited from other genera of the tribe by a base chromosome number of x = 18 (vs 11), and a combination of (usually) entire leaf margins, fasciculate inflorescences, paired bracteoles borne at the apex of the pedicel, anthers with terminal tubules but with neither spurs nor disintegration tissue, and a capsule surrounded by an accrescent, fleshy calyx, or rarely a berry. Molecular studies strongly support the monophyly of the genus, and place it as phylogenetically nested within *Gaultheria* Kalm ex L. in the Wintergreen Group clade (Powell and Kron 2001; Bush et al. 2009; Fritsch et al. 2011).

Nine species of *Diplycosia* are currently recognized in the Philippines, three having been described since the taxonomic treatment of Ericaceae for the *Flora Malesiana* (Sleumer1967; Ferreras and Argent 2011; Argent 2013; Fritsch and Amoroso in press). Because species of *Diplycosia* are often restricted to a single mountain or very few locations, more Philippine species of this genus likely await discovery (Argent 2013). Five species of *Diplycosia* have been documented from the island of Mindanao (Pelser et al. 2011 onwards; Fritsch and Amoroso in press), the southernmost major island of the Philippines. In April–May 2014 a joint botanical expedition was undertaken by the California Academy of Sciences and the CEBREM Office of Central Mindanao University to mountain peaks in the central part of Mindanao. Several individuals of *Diplycosia* were observed on Mt. Apo, the highest peak in the Philippines at 2954 m a.s.l. These plants appeared not to match any other Philippine species of the genus, according to the key in Argent (2013). Further study of a collection and photographic images made from these plants confirmed that these individuals represent a species new to science, which is here described and illustrated.

## Methods

Plants here described as a new species were dried as herbarium specimen vouchers. Macromorphological characters derived from both the specimens and photographic images of the living material were compared with those from the relevant literature sources (Sleumer 1967; Argent 1982, 1989, 2002, 2013, 2014; Ferreras and Argent 2011), specimens that are available at BRIT, CAS, and E, and online type images accessed from JSTOR Global Plants (https://plants.jstor.org/). We focused in particular on the characters used for the most recent key to Philippine *Diplycosia* in Argent (2013). Acronyms of herbaria follow Thiers (2016). Conservation status was assessed in accordance with IUCN Standards and Petitions Subcommittee (2016) criteria. The mature fruit was lost in transit between collection and deposition, and thus was not preserved; its description is based on a poor-quality photograph where only the general features were documented.

## Taxonomy

### Diplycosia platyphylla

Taxon classificationPlantaeEricalesEricaceae

P.W.Fritsch sp. nov.

urn:lsid:ipni.org:names:60472845-2

Figs 1 , 2

#### Diagnosis.

Haec species *Diplycosia urceolatae* simillima, sed ab eo petiolo viridi vel viridi ex roseo 4–7 mm longo, lamina 7–7.5 cm lata, lobis calycis acutis, calyce fructus maturo lavandulo differt.

#### Type.

PHILIPPINES. Mindanao Island, North Cotabato Province, Barangay Ilomavis, Mount Apo Natural Park, Matingao River Watershed, road from Site C to Site H at Energy Development Corporation (EDC), 1718 m, 6°59.88498'N, 125°14.86668'E, 29 Apr 2014, *D.S. Penneys 2302* (holotype: PNH!; isotypes: CAS-1196249!, CMUH-00010804!).

#### Description.

Terrestrial erect shrublet to 0.6 m tall with ascending-erect branchlets. Young branchlets not observed; old branchlets gray, stout (3.5–4.5 mm wide), subterete, with non-peeling epidermis, glabrous. Leaves spirally arranged, with distinct wintergreen odor when crushed (fresh leaves), ascending; petiole green or slightly flushed pink, 4–7 × 2.5–2.9 mm, grooved above, glabrous; lamina broadly obovate to subrotund, the larger 8.3–9.7 × 7–7.5 cm, 1.2–1.7 times as long as wide, coriaceous, abaxially glossy and evenly dark red-dotted over the surface, adaxially shiny and glabrous; major veins prominent abaxially, sulcate adaxially, major secondary veins originating on proximal half, 1 to 3 on each side of midvein, arching-ascending, alternate, occasionally 3 on one side and only 2 on the other; base broadly cuneate, margin entire, revolute, glabrous, apex rounded or strongly emarginate, the very tip with a thick protruding dark gland to 0.8 mm long. Inflorescences axillary or on older sections of defoliated branchlets proximal to the leaves, fasciculate, 3- to 6-flowered, often with up to 7 additional old fruiting pedicels; bracts ovate-deltoid, 1.5–2 × 1.2–1.7 mm, appressed-puberulent. Pedicels slightly dilated distally, 4–7 × 0.5–0.8 mm, muriculate with short (to 0.16 mm long) ferrugineous erect or ascending trichomes, and also pubescent with pale ferrugineous irregularly oriented straight to slightly undulate nonglandular trichomes to 0.3 mm long; bracteoles ovate-deltoid, 1–1.5 mm long, muriculate, rarely also sparsely puberulent along midvein, midvein planar to prominent, margin ciliolate with a mix of ferrugineous glandular trichomes and pale ferrugineous nonglandular trichomes. Calyx green strongly flushed deep pink, 2.2–2.7 × 3.4–3.7 mm, sparsely muriculate or glabrous; limb ca. 2 mm long; lobes broadly deltoid-ovate, 1.1–1.2 × 1.8–2 mm, margins with a mixture of ferrugineous glandular and pale ferrugineous nonglandular trichomes, the latter more prevalent distally, apex acute. Corolla 5-lobed, white slightly flushed with dull pink distally, broadly urceolate, widest at middle, ca. 4.5 × 2.9 mm, glabrous both outside and within; lobes recurved, ovate, ca. 1 × 1 mm, margins eciliolate, apices obtuse. Stamens 10, included within corolla, ca. 4.5 mm long; filaments ± S-curved, ca. 3 mm long, glabrous; anthers 1.7–1.9 mm long, thecae 1.4–1.5 mm long, echinulate, tubes parallel, 0.3–0.4 mm long, smooth, pores strongly oblique. Nectary 10-lobed; lobes emarginate. Ovary cylindric-hemispherical, ca. 1.5 × 1.5 mm. Style 2.5–3 mm long, glabrous. Immature fruit green flushed red, 5–6 × 5–6 mm, style persistent; mature fruiting calyx lavender, accrescent, fleshy.

**Figure 1.**
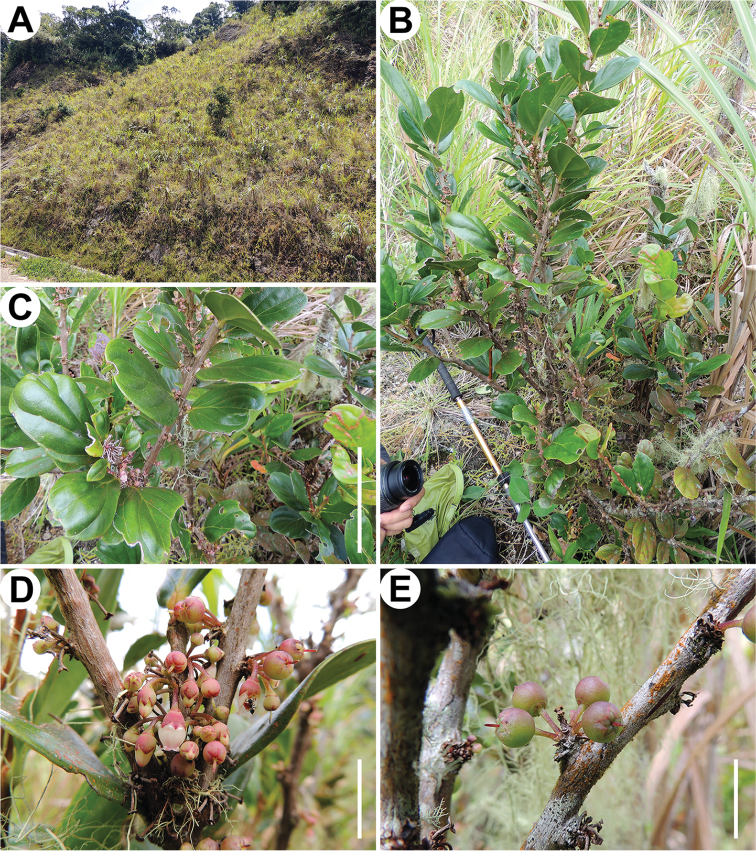
*Diplycosia platyphylla* P.W.Fritsch. **A** Habitat **B** Habit **C** Branchlet with inflorescences **D** Inflorescence **E** Immature fruit. (D.S. Penneys 2302). Scale bars: **C** = 10 cm, **D**, **E** = 1 cm. Photos P.W.F.

**Figure 2.**
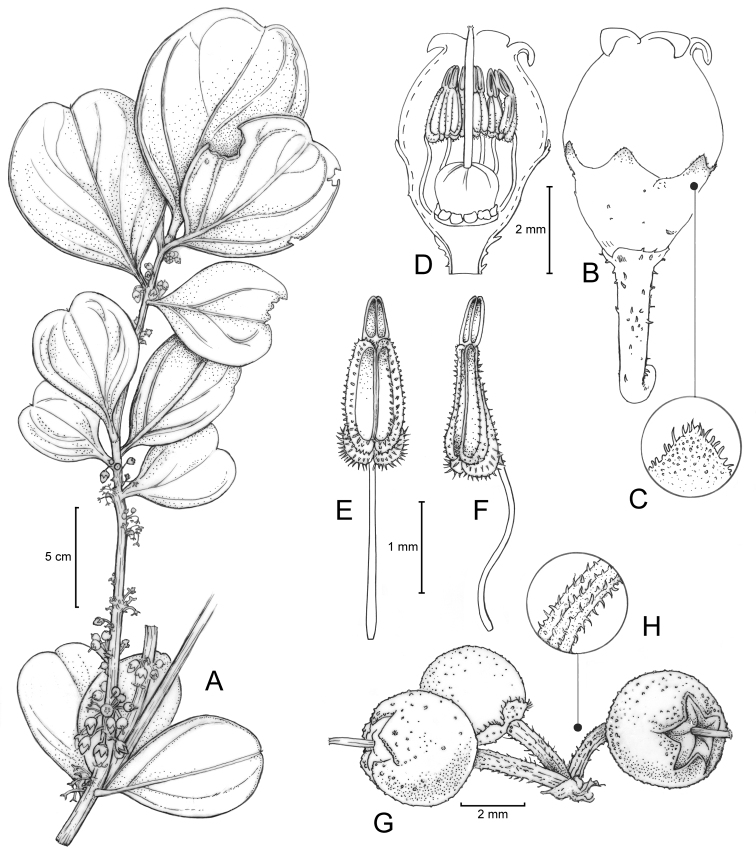
*Diplycosia platyphylla* P.W.Fritsch. **A** Fertile branchlet **B** Pedicel and flower **C** Calyx lobe apex **D** Flower with calyx and corolla partly cut away to show androecium and gynoecium **E** Stamen, ventral view **F** Stamen, lateral view **G** Immature fruit **H** Fruiting pedicel. Drawing by L. Heagy from D.S. Penneys 2302 (CAS) and photographic images of the living plant.

#### Etymology.

The species is named for its notably wide leaves relative to those of most other species in the genus.

#### Phenology.

Flowering and fruiting in April.

#### Distribution and habitat.

*Diplycosia platyphylla* is known only from a single location on Mt. Apo, Mindanao Island, North Cotabato Province, Philippines. Several individuals were found growing in gravel on an ultramafic open sunny rockslide area in the Tropical Lower Montane Rain Forest biome at 1718 m a.s.l. with *Nepenthes* L., *Huperzia* Bernh., and *Vaccinium* L. on a general NW-facing 30–60% slope but in a flat microhabitat along a roadside.

#### Conservation status.

*Diplycosia platyphylla* is known from a single population and single collection, with only several plants seen. Although the species is afforded protection by its occurrence in Mount Apo National Park, it is at risk through apparent extreme rarity. We categorize this species as Critically Endangered (CR): D.

#### Discussion.

The new species is similar to *Diplycosia urceolata* Stapf from Mount Kinabalu in Borneo, Sabah, Malaysia by its stout glabrous branchlets, coriaceous glabrous leaf blades with rounded apices, and 4–7 mm-long pedicels with a mixture of muriculate trichomes and pale ferrugineous nonglandular trichomes. It differs from this species most readily by its green or slightly flushed pink petioles 4–7 mm long (vs vivid red and 10–13 mm), wider leaf blades [7–7.5 cm vs (2.5–)3.5–5(–7.5) cm], acute calyx lobe apices (vs obtuse), and lavender mature fruiting calyx (vs black). It is also similar to *Diplycosia sanguinolenta* Sleumer from Mount Kinabalu in its large coriaceous leaf blades [9–14(–17) × 5–8(–9.5) cm for *Diplycosia sanguinolenta*; Sleumer 1967] and generally acute calyx lobes, but is easily distinguished by a petiole 4–7 mm long (vs 12–15 mm) and a white corolla ca. 4.5 mm long (vs bright red and 13–15 mm long), among other characters. The new species would key to couplet 3 of the key to the Philippine *Diplycosia* in Argent (2013). The two leads at that point in the key identify the varieties of *Diplycosia luzonica* (A.Gray) Merrill, i.e., Diplycosia luzonica var. calelanensis (Elmer) Sleumer and Diplycosia luzonica var. merrittii (Merrill) Sleumer, distinguished by “Leaves broadly pointed to rounded with the terminal gland forming a short mucronate point the largest leaves up to 5 cm long” vs “Leaves acuminate, with a narrowly acute apex, the largest leaves more than 6 cm long,” respectively. Because the new species possesses leaves that are rounded (or strongly emarginate) at the apex but are 8.3–9.7 cm long (the larger ones), the new species does not match either of these leads. Thus, it can be distinguished from all other Philippine *Diplycosia* by the combination of glabrous branchlets and (larger) leaf blades 8.3–9.7 mm long with an apex that is rounded or strongly emarginate, and with a prominent terminal gland.

## Supplementary Material

XML Treatment for Diplycosia platyphylla
